# *NewWave*: a scalable R/Bioconductor package for the dimensionality reduction and batch effect removal of single-cell RNA-seq data

**DOI:** 10.1093/bioinformatics/btac149

**Published:** 2022-03-10

**Authors:** Federico Agostinis, Chiara Romualdi, Gabriele Sales, Davide Risso

**Affiliations:** Department of Biology, Università degli Studi di Padova, Padova 35100, Italy; Department of Biology, Università degli Studi di Padova, Padova 35100, Italy; Department of Biology, Università degli Studi di Padova, Padova 35100, Italy; Department of Statistical Science, Università degli studi di Padova, Padova 35100, Italy

## Abstract

**Summary:**

We present *NewWave*, a scalable R/Bioconductor package for the dimensionality reduction and batch effect removal of single-cell RNA sequencing data. To achieve scalability, *NewWave* uses mini-batch optimization and can work with out-of-memory data, enabling users to analyze datasets with millions of cells.

**Availability and implementation:**

*NewWave* is implemented as an open-source R package available through the Bioconductor project at https://bioconductor.org/packages/NewWave/

**Supplementary information:**

Supplementary data are available at *Bioinformatics* online.

## 1 Introduction

Dimensionality reduction is a key step for the analysis of single-cell RNA-seq (scRNA-seq) data. Principal component analysis (PCA) is a simple and efficient method that can be employed for this step. However, it suffers from several drawbacks, e.g. it assumes that the data are Gaussian and does not allow to correct for technical variability and biases. While transforming the data (e.g. by running PCA on log-normalized counts) can ameliorate these problems, count-based factor analysis models often yield better low-dimensional data representations (Risso *et al.*, 2018; Townes *et al.*, 2019).

In particular, our recent method, ZINB-WaVE (Risso *et al.*, 2018), uses a zero-inflated negative binomial model to find biologically meaningful latent factors. Optionally, the model can remove batch effects and other confounding variables (e.g. sample quality), leading to a low-dimensional representation that focuses on biological differences among cells.

ZINB-WaVE has been shown to be among the top performing methods in recent benchmarks (Raimundo *et al.*, 2020; Sun *et al.*, 2019). However, its main drawback is the lack of scalability, due to large memory requirements that prevent its use with more than a few cores. To address this, we have reimplemented the model of ZINB-WaVE in a new Bioconductor package, *NewWave*, which allows users to massively parallelize computations using PSOCK clusters. Here, we show that *NewWave* is able to achieve the same, or even better, performance of ZINB-WaVE at a fraction of the computational speed and memory usage, reducing the runtime by 90% with respect to ZINB-WaVE.

## 2 Software implementation

**Fig. 1.**
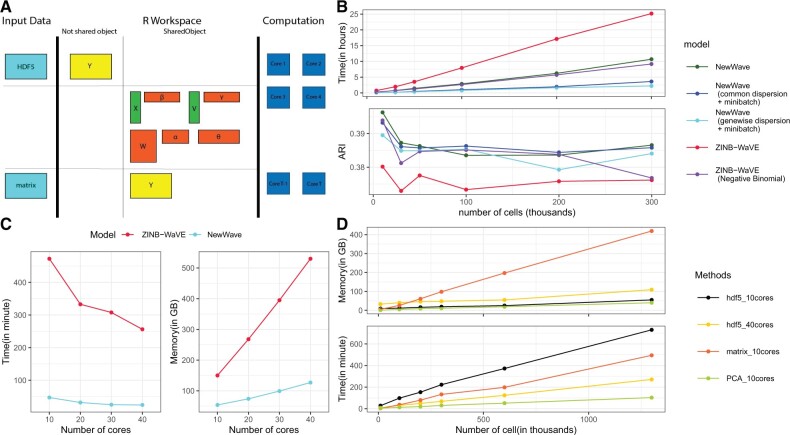
Implementation and performance of *NewWave*. Unless otherwise noted, we used 10% of the observations as the size of the mini-batches and 10 cores. (**A**) Schema of the *NewWave* model, indicating which matrices are in shared memory (see Supplementary Information for more details). (**B**) Speed (top) and ARI (bottom) of *NewWave* (in-memory data) with different choices of the parameters and ZINB-WaVE applied to the BICCN dataset (Yao *et al.*, 2021) with a maximum of 312 000 cells and after selecting the 1000 most variable genes. The reported ARI is computed as the mean ARI of 100 *k*-means clustering procedures with the number of centroids set to the known number of labels (*k* = 20). (**C**) Speed and RAM usage of *NewWave* (gene-wise dispersion + mini-batch) and ZINB-WaVE using a subset of 100 000 cells varying the number of cores used for computation. (**D**) RAM usage (top) and speed (bottom) of *NewWave* on the 10X 1.3 M cell datasets with 1000 most variable genes

*NewWave* uses a factor analysis framework similar to that of ZINB-WaVE (Risso *et al.*, 2018), with the important difference that the gene-level read counts are assumed to come from a negative binomial distribution without zero inflation. In fact, the majority of large scRNA-seq data use unique molecular identifiers (UMIs) and UMI data are not zero inflated (Svensson, 2020; Townes *et al.*, 2019). Briefly, the log of the expected value of the read count matrix is modeled as a regression of three terms: known cell covariates (*X*, e.g. batch), known gene covariates (*V*, e.g. an intercept with the role of normalization) and latent factors (*W*) that define a low-dimensional space that describe the unknown biological signal (Fig. 1A and Supplementary Information). With a high number of cells, these matrices are large and it may not be easy to control how many times they are copied during parallel execution.

The three main strategies that *NewWave* uses to limit the computational problems of working with large matrices are: (i) the use of shared memory objects in PSOCK clusters to avoid redundant data copies, (ii) the use of mini-batch optimization algorithms to speed-up computations and (iii) the use of out-of-memory data representations (such as HDF5 files) to limit memory usage.

The optimization procedure can be represented as a cycle of three steps, iterated until convergence: (i) optimization of the dispersion parameters (either common dispersion or gene-wise dispersion); (ii) optimization of gene-wise parameters and (iii) optimization of cell-wise parameters.

One of the main advantages of our model specification is that it naturally results in an embarrassingly parallel task. In fact, except for the optimization of the global dispersion parameter (common to all genes), all the steps use only one gene (cell) at a time for the optimization of gene (cell) parameters. In addition to parallelization, this setup is ideal for mini-batch optimization strategies. At any one step, we can use a random subset of cells (genes) to estimate the gene (cell) parameters.

On-disk datasets are managed through the *DelayedArray* package (Pagès *et al.*, 2021), which allows block processing and delayed operations on data stored in HDF5 files. While all covariates and parameter matrices are stored in shared memory among child processes, the input data can reside either in shared memory or on-disk as an HDF5 file (Fig. 1A).

## 3 Results and discussion

The application of *NewWave* to subsamples of large datasets, in particular when relying on mini-batches, shows a better scalability than ZINB-WaVE without loss of accuracy (Fig. 1B; see Supplementary Information for details on the analysis). We benchmarked the accuracy of *NewWave* against ZINB-WaVE in terms of Adjusted Rand Index (ARI) and Akaike Information Criterion (see Supplementary Information for details on the evaluation metrics).

Strikingly, the negative binomial model outperforms its zero-inflated counterpart, confirming that this is a preferable model for UMI data (Fig. 1B and Supplementary Table S1). To evaluate the ability of *NewWave* to remove unwanted variation, we applied it to two datasets with known batch effects and showed that it leads to a good mix of batches and a good separation among putative cell types (Supplementary Figs S1 and S2).

In addition to speed, we measured the scalability of *NewWave* in terms of RAM usage (Fig. 1C and D). As expected, there is a speed-RAM trade-off when using data in-memory or on-disk. Runtimes increase when using HDF5, due to the additional I/O, but this dramatically decreases the RAM consumption. This in turn allows the use of more cores. Using 40 cores, the computational time of our HDF5 implementation is lower than that of the in-memory data with 10 cores, allowing us to analyze 1.3 M cells in 271 min using 109 GB of RAM (Fig. 1D).

*NewWave* is available as an open-source package through the Bioconductor project. The package includes a vignette with a tutorial. In addition, the code to reproduce all the analyses presented here is available at https://github.com/fedeago/NewWave-script.

Future work will be focused on leveraging sparse matrix formats, either in-memory or on-disk, e.g. through the *TileDB* format, to speed-up data access and computations.

## Funding

This work was supported by the AIRC Foundation for Cancer Research in Italy [AIRC 21837 to C.R.]; the National Cancer Institute of the National Institutes of Health [2U24CA180996 to D.R.]; the Chan Zuckerberg Initiative DAF [CZF2019-002443 to D.R.] an advised fund of Silicon Valley Community Foundation and by University of Padova Strategic Research Infrastructure Grant 2017: ‘CAPRI: Calcolo ad Alte Prestazioni per la Ricerca e l’Innovazione’.

*Conflict of Interest*: none declared.

## Data availability

All datasets used in this paper are publicly available. The BICCN data can be downloaded from http://data.nemoarchive.org/biccn/lab/zeng/transcriptome/. The 10X Brain data are available through the TENxBrainData package available at https://bioconductor.org/packages/TENxBrainData. The RNA mixture data are available at https://github.com/LuyiTian/sc_mixology/.

## Supplementary Material

btac149_Supplementary_Data
