# Supplementary material for: NewWave: a scalable R/Bioconductor package for the dimensionality reduction and batch effect removal of single-cell RNA-seq data
Source: Bioinformatics. 2022 Mar 10;38(9):2648–50. doi: 10.1093/bioinformatics/btac149 (PMC9048694; doi:10.1093/bioinformatics/btac149)
Supplement: btac149_Supplementary_Data [file btac149_supplementary_data.pdf]

Supplementary material for NewWave: a scalable R/Bioconductor package for the dimensionality reduction and batch effect removal of single-cell RNA-seq data

Federico Agostinis, Chiara Romualdi, Gabriele Sales, Davide Risso

March 4, 2022

1 Model specification

NewWave assumes a negative binomial distribution for the expression data. Given n samples and J genes across all samples and denoting by Y_{ij} the count of gene j in cell i, the likelihood function for the observed count y_{ij} is

$$f_{NB}(y_{ij};\mu_{ij},\theta_j) = \frac{\Gamma(y_{ij}+\theta_j)}{\Gamma(y_{ij}+1)\Gamma(\theta_j)}\left(\frac{\theta_j}{\theta_j+\mu_{ij}}\right)^{\theta_j}\left(\frac{\mu_{ij}}{\mu_{ij}+\theta_j}\right)^{y_{ij}} \tag{1}$$

in this parametrization the variance is

$$\sigma^2_{ij} = \mu_{ij} + \frac{\mu_{ij}}{\theta_j} = \mu_{ij} + \phi\mu_{ij} \tag{2}$$

We specify the following regression model:

$$\ln(\mu_{ij}) = \left(X\beta + (V\gamma)^T + W\alpha\right)_{ij}, \tag{3}$$

where X is a matrix with dimension $n \times M$ where M is the number of cell-level covariates. It typically contains a set of dummy variables that specify the batch of the samples and by default includes an intercept. V is a matrix with dimension $J \times L$ where L is the number of gene-level covariates. It typically only contains an

intercept, used as library size normalization. W is a matrix with dimension $n \times K$, where K is the number of latent factors, and it contains the low-rank representation of the cells. The parameters α, β, and γ are the regression coefficients for W, X, and W, respectively.

We note that (3) is a special case of the model of Risso et al. (2018) and we refer the reader to that paper for additional details.

1.1 Parameter estimation

The log-likelihood function to be maximized is

$$l(\beta, \gamma, W, \alpha, \theta) = \sum_{i=1}^{n} \sum_{j=1}^{J} \ln f_{NB}(Y_{ij}; \mu_{ij}, \theta_j) \tag{4}$$

The estimation of the parameters is done with a penalized approach for β, α, W, γ and not penalized for θ:

$$\max_{\beta,\gamma,W,\alpha,\zeta} l(\beta, \gamma, W, \alpha, \theta) - Pen(\beta, \gamma, W, \alpha), \tag{5}$$

where $Pen(\cdot)$ is a regularization term to reduce overfitting and improve numerical stability (see Risso et al., 2018, for details).

Our iterative estimation procedure follows closely that of Risso et al. (2018), considering the special case of no zero inflation.

Special attention must be paid to the estimation of the dispersion parameters θ_j. These can be estimated in a gene-wise fashion, or assuming a common dispersion parameter $\theta_j = \theta$ for all the genes.

This choice yields two different implementations. When the user wants to estimate a common dispersion parameter, our implementation takes advantage of the mini-batch strategy outlined below. However, the computation cannot leverage parallel computations, since only one value needs to be estimated. On the other hand, when the user requires gene-wise dispersion parameters, the computations can proceed in parallel for each gene, but the mini-batch strategy is not effective, as it slows down computations.

1.2 Mini-batches

Even when the mini batch approach is chosen, the first iteration will be done using all the observation; this method shows better performance in terms of time

needed to converge. If the common dispersion approach is chosen, starting from the second iteration, only a random subset b of size m of observations is used at each iteration to estimate the parameter. Note that the parameter estimate is assumed to describe the variation across all observations, even those that do not belong to b.

At each iteration, the estimated parameter is optimal for b, but there is no guarantee of its optimality on the full data. Hence, the value of the parameter estimate is updated only if it leads to an increase in the log-likelihood function in the full data.

The optimization of the cell- and gene-specific parameters is performed with a mini-batch approach after a first iteration that uses all data. When parallel computing is used, each child process uses only m/c observations where m is the number of observations in the mini-batch and c is the number of child processes.

2 Details on the benchmark

2.1 Compared strategies

Figure 1B shows a benchmark of the different estimation strategies implemented in the *NewWave* package, compared to the original ZINB-WaVE implementation, both with and without zero inflation.

The estimation strategies in *NewWave* are

- **Default (Common dispersion, no mini-batches)**. A single dispersion parameter, common to all genes, is estimated. All cells and genes are used for the estimation of gene- and cell-specific parameters.
- **Common dispersion + mini-batch**. A single dispersion parameter, common to all genes, is estimated. Mini-batches of 10% of the number of cells and 100 genes are used for the estimation of gene- and cell-specific parameters, respectively.
- **Gene-wise dispersion + mini-batch**. Gene-wise dispersion parameters are estimated. Mini-batches of 10% of the number of cells and 100 genes are used for the estimation of gene- and cell-specific parameters, respectively.

Note that since our preprocessing includes a preliminary step that selects the top 1,000 most variable genes, the genes in the minibatches are a random sample of these.

Figure 1D shows a benchmark of different specifications of the *NewWave* model:

- **hdf5_10cores** : NewWave with minibatch and genewise dispersion on a out-of-memory dataset (HDF5 format) using 10 cores.
- **hdf5_40cores** : NewWave with minibatch and genewise dispersion on a out-of-memory dataset (HDF5 format) using 40 cores.
- **matrix_10cores** : NewWave with minibatch and genewise dispersion on a dense in-memory matrix using 10 cores.
- **PCA_10cores** : Exact PCA on a out-of-memory dataset (HDF5 format) using 10 cores. We used exact PCA rather than randomized PCA as our preliminary results suggested that the former was faster.

2.2 Datasets used

We used three publicly available datasets for our benchmark, namely the BICCN dataset, the 10X Brain dataset, and the RNA mixture dataset.

BICCN data. We created the first dataset starting from the mouse primary motor cortex datasets generated by the BRAIN Initiative Cell Census Network (BICCN) (Yao et al., 2021), which we refer to as the BICCN dataset.

The raw data was generated as part of the BICCN consortium and can be downloaded from `http://data.nemoarchive.org/biccn/lab/zeng/transcriptome/`.

The known batch effect present in this dataset is due to the difference between platforms, namely 10X Chromium single-cell sequencing v2 and v3 and 10X Chromium single-nucleus sequencing.

We selected the 1,000 most variable genes after correcting for the batch effects using the mutual nearest neighbor method (**?**), as implemented in the *fastMNN* function of the *batchelor* Bioconductor package (v. 1.3.14). All the methods were applied to the raw counts of these 1,000 genes.

10X Brain data. The second dataset is the 1.3 million brain cell single-cell RNA-seq (scRNA-seq) data set generated by 10X Genomics.

The dataset is available in dense HDF5 format as part of the *TENxBrainData* Bioconductor package at `https://bioconductor.org/packages/TENxBrainData`. We selected the 1,000 most variable genes on the entire dataset.

RNA mixture data This dataset is made of 636 samples obtained from a mixture of RNA from three different cell lines (see Tian et al., 2019, for details). Samples were obtained using two different technologies that represent the batch effect.

2.3 Evaluation strategy

Here, we briefly describe the two metrics used to evaluate the accuracy of the compared methods, namely the Adjusted Rand Index (ARI) and the Akaike Information Criterion (AIC).

2.3.1 Adjusted Rand Index (ARI)

The ARI is a similarity measure between two partitions (usually a prediction and a ground truth). It is the adjusted version of the Rand Index (RI) (Hubert and Arabie, 1985). RI considers all pairs of samples and counts pairs that are assigned in the same or different clusters respectively in the predicted and true clusterings. Given a set S and two partitions of S, partition X composed of r subsets and partition Y composed of s subsets we define the following:

- a, the number of pairs of elements in S that are in the same subset in X and in the same subset in Y
- b, the number of pairs of elements in S that are in different subsets in X and in different subsets in Y
- c, the number of pairs of elements in S that are in the same subset in X and in different subsets in Y
- d, the number of pairs of elements in S that are in different subsets in X and in the same subset in Y

Then the RI is defined as:

$$RI = \frac{a+b}{a+b+c+d}$$

.

The raw RI is then "adjusted for chance" into the ARI score using the following formula:

$$ARI = \frac{RI - Expected(RI)}{max(RI) - Expected(RI)}$$

The expected value is estimated using a permutation approach where the number and size of clusters within a clustering are fixed, and all random clusterings are generated by shuffling the elements between the fixed clusters.

The ARI has a value close to 0 for random labeling and 1 when the clusterings are identical (up to a permutation).

2.3.2 Akaike Information Criterion (AIC)

The AIC is a mathematical method for evaluating how well a model fits the data. It is used to compare different models and determine which one is the best fit for the data. The AIC can be interpreted as the value of the log-likelihood "adjusted" for the number of parameters of the model. In our case, the AIC can be written as:

$$AIC = 2k - 2l(\hat{\beta}, \hat{\gamma}, \hat{W}, \hat{\alpha}, \hat{\theta}),$$

where $k = J(M + K + 1) + n(L + K)$ is the total number of parameters and $\hat{\beta}$, $\hat{\gamma}$, $\hat{W}$, $\hat{\alpha}$, and $\hat{\theta}$ are the estimates of the parameters of the model.

The AIC is a relative measure: its value cannot be interpreted in absolute terms, but can only be used to compare models. In particular, the lower the AIC the better the model. The best model according to AIC is the one that explains the greatest amount of variation using the fewest possible parameters.

3 Batch effect reduction

In this section we report additional results on the ability of *NewWave* to reduce batch effects. To do so we employ two datasets: the BICCN dataset, which is a complex real dataset, but lacks ground truth, and the RNA mixture, which has the advantage of providing ground truth labels.

3.1 BICCN dataset

Supplementary Figure 1 shows a UMAP representation of the full BICCN data color-coded by batch (panel A, B) and putative cell type (panel C, D).

Supplementary Figure 1 shows that NewWave reduces batch effects and leads to a better clustering of cell types compared to PCA.

3.2 RNA mixture dataset

The analysis of the RNA mixture data leads to similar conclusions. Briefly, the RNA mixture data is derived from seven mixtures of three cell lines (HCC827, H1975 and H2228) in different proportions, sequenced with two different technologies. Here, the single-cell protocol (CEL-seq2 vs SORT-seq) is considered a batch effect and the seven mixtures as the biological groups of interest.

Supplementary Figure 2 shows that NewWave is able to correct the batch effect generated from the different technologies used. In particular, the two batches are well mixed (panel A) and the proportions are correctly ordered in the UMAP plot (panel C).

In order to quantify the batch effect removal we performed a cluster analysis using the Louvain algorithm. The partition obtained after applying NewWave results in an ARI of 0.60 while the partition obtained with the PCA workflow results in an ARI of 0.50.

4 Acknowledgements

The authors thank Hongkui Zeng and the members of the BICCN consortium for sharing the BICCN dataset.

5 Supplementary Tables and Figures

Table 1: Time, Akaike Information Criterion (AIC), and Adjusted Rand Index (ARI) for the different dimentionality reduction methods on the BICCN dataset. This table reports the data displayed in Fig. 1B.

Model	# of cells	Time in minutes	AIC	ARI
NewWave	10000	15.64	39097804	0.3963
NewWave (common dispersion + minibatch)	10000	5.67	39177295	0.3932
NewWave (genewise dispersion + minibatch)	10000	3.96	38933804	0.3895
ZINB-WaVE	10000	44.06	43068497	0.3802
ZINB-WaVE (Negative Binomial)	10000	16.24	50010784	0.3939
NewWave	30000	45.26	116840934	0.3873
NewWave (common dispersion + minibatch)	30000	10.30	117173555	0.3861
NewWave (genewise dispersion + minibatch)	30000	11.32	116363662	0.3849
ZINB-WaVE	30000	117.27	128899425	0.3730
ZINB-WaVE (Negative Binomial)	30000	44.33	149574224	0.3812
NewWave	50000	85.92	194632320	0.3863
NewWave (common dispersion + minibatch)	50000	27.80	195018975	0.3857
NewWave (genewise dispersion + minibatch)	50000	21.24	193863319	0.3849
ZINB-WaVE	50000	211.62	214638707	0.3775
ZINB-WaVE (Negative Binomial)	50000	74.80	249048748	0.3847
NewWave	100000	171.48	389698453	0.3835
NewWave (common dispersion + minibatch)	100000	60.29	390483587	0.3863
NewWave (genewise dispersion + minibatch)	100000	47.12	388019619	0.3853
ZINB-WaVE	100000	476.28	430023667	0.3733
ZINB-WaVE (Negative Binomial)	100000	158.59	498933752	0.3851
NewWave	200000	372.64	780334158	0.3836
NewWave (common dispersion + minibatch)	200000	116.18	782011394	0.3844
NewWave (genewise dispersion + minibatch)	200000	98.73	777041944	0.3793
ZINB-WaVE	200000	1027.81	861598414	0.3758
ZINB-WaVE (Negative Binomial)	200000	343.35	999580927	0.3838
NewWave	300000	641.30	1211144264	0.3866
NewWave (common dispersion + minibatch)	300000	216.55	1213490638	0.3858
NewWave (genewise dispersion + minibatch)	300000	131.99	1205982980	0.3841
ZINB-WaVE	300000	1511.18	1338439179	0.3762
ZINB-WaVE (Negative Binomial)	300000	550.61	1551562402	0.376

Table 2: Time and RAM usage of NewWave and ZINB-WaVE varying the number of CPUs. This table reports the data displayed in Fig. 1C.

**Model**	**Time in minutes**	**# of cores**	**Memory in GB**
NewWave	46.97	10	54
NewWave	31.69	20	74
NewWave	25.00	30	99
NewWave	24.22	40	127
ZINB-WaVE	472.53	10	150
ZINB-WaVE	332.94	20	268
ZINB-WaVE	307.83	30	395
ZINB-WaVE	256.19	40	530

Table 3: Time and RAM usage of NewWave on the 1.3 million cell dataset. This table reports the data displayed in Fig. 1D.

Model	# of cells	RAM in GB	Time in minutes
hdf5_10cores	10000	9	28.364
hdf5_40cores	10000	33	9.063
matrix_10cores	10000	5	3.768
PCA_10cores	10000	1	5.452
hdf5_10cores	100000	12	98.240
hdf5_40cores	100000	40	31.081
matrix_10cores	100000	26	36.611
PCA_10cores	100000	5	13.375
hdf5_10cores	200000	16	153.370
hdf5_40cores	200000	46	49.374
matrix_10cores	200000	61	79.583
PCA_10cores	200000	9	19.562
hdf5_10cores	300000	19	222.784
hdf5_40cores	300000	48	68.381
matrix_10cores	300000	98	132.788
PCA_10cores	300000	11	30.691
hdf5_10cores	600000	25	372.509
hdf5_40cores	600000	55	124.352
matrix_10cores	600000	197	198.116
PCA_10cores	600000	19	51.960
hdf5_10cores	1300000	55	730.297
hdf5_40cores	1300000	109	271.085
matrix_10cores	1300000	419	494.515
PCA_10cores	1300000	40	102.940

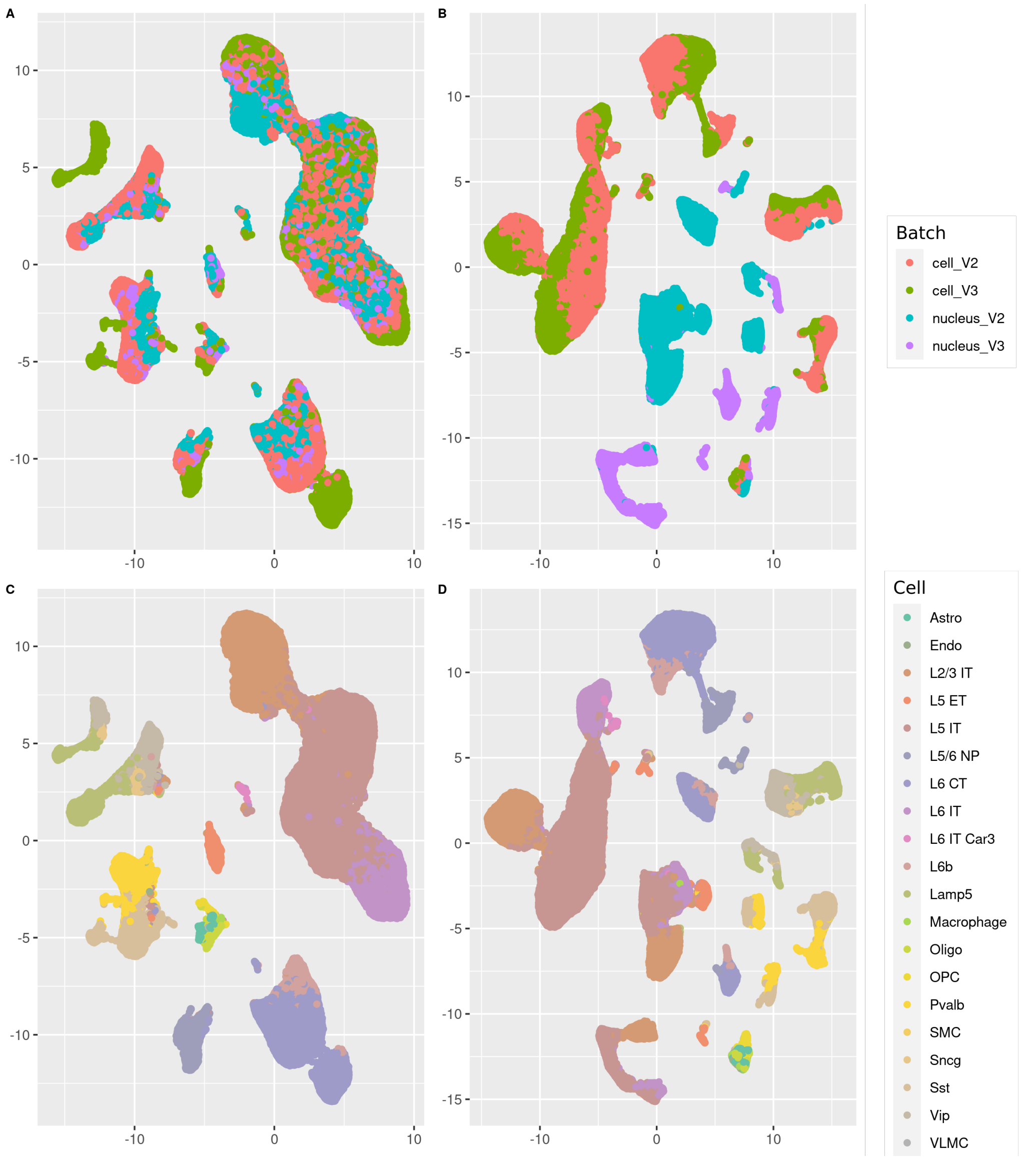

Figure 1: **Comparison between NewWave and PCA in the BICCN dataset**. **A**. UMAP following NewWave dimensionality reduction and color coded by batch. **B**. UMAP following PCA dimensionality reduction and color coded by batch. **C**. UMAP following NewWave dimensionality reduction and color coded by putative cell type. **D**. UMAP following PCA dimensionality reduction and color coded by putative cell type.

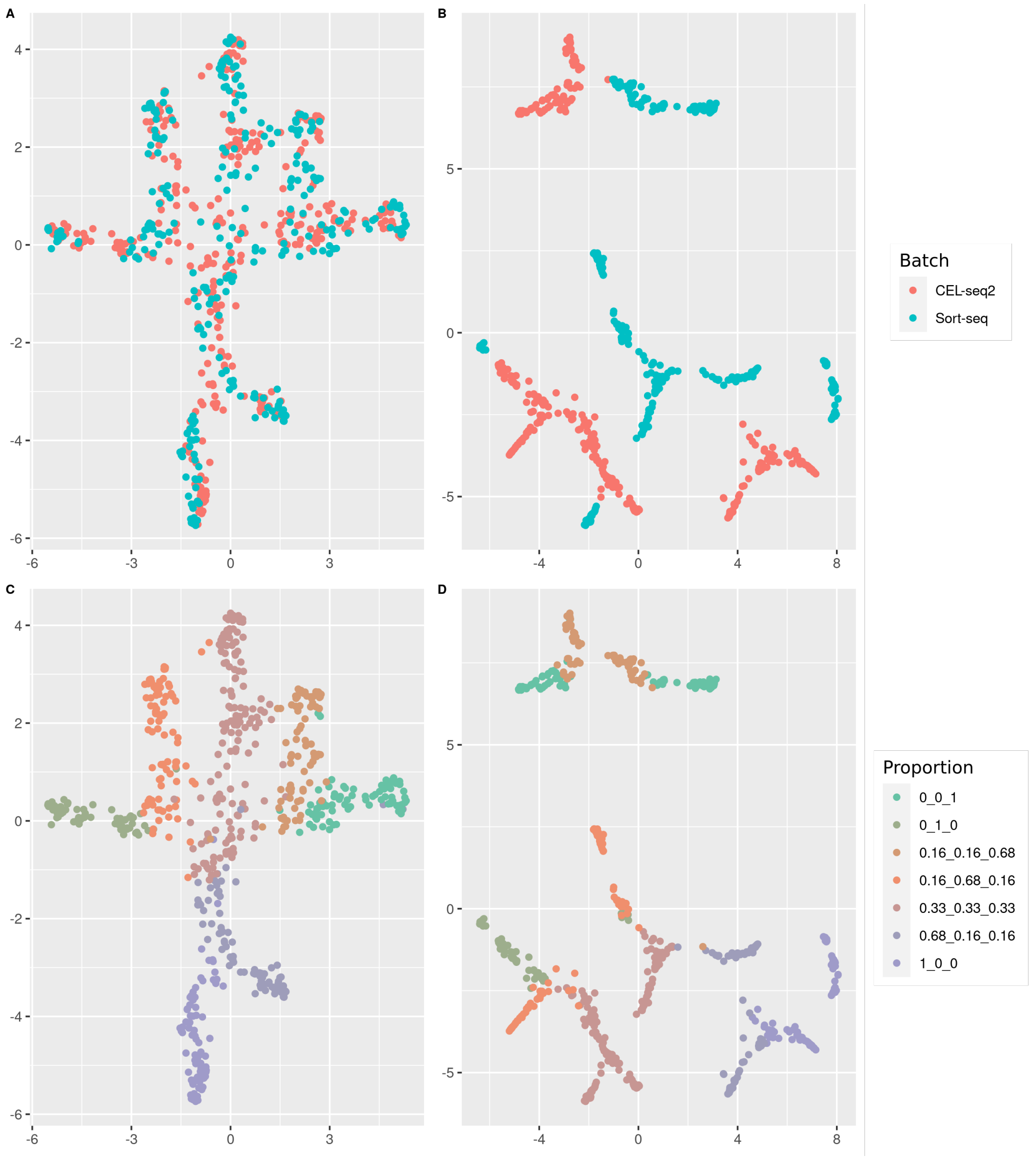

Figure 2: **Comparison between NewWave and PCA in the RNA mixture dataset**. **A**. UMAP following NewWave dimensionality reduction and color coded by batch. **B**. UMAP following PCA dimensionality reduction and color coded by batch. **C**. UMAP following NewWave dimensionality reduction and color coded by putative cell type. **D**. UMAP following PCA dimensionality reduction and color coded by putative cell type.

References

Davide Risso, Fanny Perraudeau, Svetlana Gribkova, Sandrine Dudoit, and Jean Philippe Vert. A general and flexible method for signal extraction from single-cell RNA-seq data. *Nature Communications*, 2018. ISSN 20411723. doi: 10.1038/s41467-017-02554-5.

Zizhen Yao, Hanqing Liu, Fangming Xie, Stephan Fischer, Ricky S Adkins, Andrew I Aldridge, Seth A Ament, Anna Bartlett, M Margarita Behrens, Koen Van den Berge, et al. A transcriptomic and epigenomic cell atlas of the mouse primary motor cortex. *Nature*, 598(7879): 103–110, 2021.

Luyi Tian, Xueyi Dong, Saskia Freytag, Kim-Anh Lê Cao, Shian Su, Abolfazl JalalAbadi, Daniela Amann-Zalcenstein, Tom S Weber, Azadeh Seidi, Jafar S Jabbari, et al. Benchmarking single cell rna-sequencing analysis pipelines using mixture control experiments. *Nature methods*, 16(6):479–487, 2019.

Lawrence Hubert and Phipps Arabie. Comparing partitions. *Journal of Classification*, 2(1):193–218, Dec 1985. ISSN 1432-1343. doi: 10.1007/BF01908075. URL `https://doi.org/10.1007/BF01908075`.
